# Acceptance of pharmaceutical cannabis substitution by cannabis using patients with schizophrenia

**DOI:** 10.1186/s12954-018-0253-7

**Published:** 2018-09-20

**Authors:** Jan van Amsterdam, Jojanneke Vervloet, Gerdien de Weert, Victor J. A. Buwalda, Anna E. Goudriaan, Wim van den Brink

**Affiliations:** 1 Department of Psychiatry, Academic Medical Center, University of Amsterdam, P.O. Box 22660, 1100 DD Amsterdam, The Netherlands; 2 Jellinek-Arkin, P.O. Box 75848, 1070 AV Amsterdam, The Netherlands

**Keywords:** Schizophrenia, Psychosis, Cannabis, Skunk, CBD, High-potency cannabis

## Abstract

**Background:**

Cannabis-smoking patients with a psychotic disorder have poorer disease outcomes than non-cannabis-smoking patients with poorest outcomes in patients smoking high-potency cannabis (HPC) containing high Δ9-tetrahydrocannabinol (THC) and low cannabidiol (CBD). Quitting cannabis smoking or substitution of HPC by cannabis variants containing less THC and/or more CBD may benefit these patients. The present study explores whether daily HPC-smoking patients with schizophrenia accept smoking such variants.

**Methods:**

Twelve male patients were asked to smoke on six different occasions one joint: on two occasions, the cannabis they routinely smoke (HPC; not blind), and blind in random order; on two occasions, cannabis containing low THC and no CBD; and on two occasions, cannabis containing low THC and high CBD.

**Results:**

Both substitute variants were appreciated, but patients preferred the HPC they usually smoked. The effect of the low THC/high CBD variant was reported by 32% to be too short and by 36% to be not strong enough, whereas this was reported by 5% and 64%, respectively, for the low THC cannabis variant.

**Conclusions:**

Based on these findings, a larger and longer study on the efficacy of cannabis substitution treatment in HPC-smoking patients with schizophrenia seems feasible and should be considered.

**Trial registration:**

2014-005540-17NL. Registered 22 October 2014, 2014-005540-17NL 20141215 CTA.xml

## Introduction

Prevalence of cannabis use in patients with a psychotic disorder is high [1, 2]. According to patients, cannabis is often used as self-medication to ameliorate positive and/or negative symptoms [3], to get “high,” and/or for pleasure and social motives [4–6]. However, there is growing evidence that (a) patients with a psychotic disorder who smoke high-potency cannabis (HPC), i.e., cannabis containing high concentrations of Δ9-tetrahydrocannabinol (THC) and low concentrations of cannabidiol (CBD), have poorer disease outcomes than either non-cannabis-smoking patients or patients that discontinue their cannabis use and (b) patients who continue the use of HPC show more psychotic episodes and an increased probability of (re)hospitalization [7, 8].

THC seems to be the main driver of the psychotogenic effects of cannabis, whereas CBD may inhibit THC-induced anxiety and psychosis. In a large web-based cross-sectional survey, it was shown that persons who smoked cannabis with a low THC/CBD ratio had significantly fewer psychotic-like experiences than those smoking cannabis with higher THC/CBD ratios [9]. Furthermore, co-administration of CBD and THC induced less anxiety and fewer psychotic-like symptoms than THC alone [10–14], implicating that smoking CBD-enriched cannabis (and containing less THC) may be less harmful for patients with a psychotic disorder who do not want or cannot quit smoking cannabis [13, 14]. Therefore, prescribing cannabis containing less THC and/or more CBD as a substitution for HPC is a promising form of harm reduction, i.e., allowing the use of cannabis and simultaneously reducing the risk of psychotic episodes and (re)hospitalization. In the current study, we investigated whether patients with a psychotic disorder using HPC accept smoking less harmful cannabis variants, i.e., cannabis variants with less THC and/or more CBD.

## Methods

### Study design

The cannabis that patients regularly smoked (“own cannabis”) was used as a reference to evaluate the acceptability of two medicinal cannabis variants containing less THC. In the presence of the investigator, patients smoked one joint on six separate days. The cannabis (0.25 g) was rolled in tobacco. The first and last joint contained the patient’s own cannabis. The other four joints were offered in a blinded and random order and contained either 0.25 g of Bediol® (3.6% *w*/*w* THC and 7.1% *w*/*w* CBD; two times) or 0.25 g of Bedrobinol® (9.1% *w*/*w* THC and < 0.2% *w*/*w* CBD; two times). For product details about Bedrobinol and Bediol, please see the website of the Office Medicinal Cannabis (BMC) [15]. The patient’s own cannabis was assayed for THC and CBD.

### Inclusion criteria

Twelve male outpatients (mean 40.5 ± 10.0 years; range 22–54 years.) who were in treatment for schizophrenia and a comorbid cannabis use disorder were included. All were daily cannabis smokers, currently treated with antipsychotics, and had suboptimal response to treatment, i.e., persistent psychotic symptoms and poor social behavior. One patient dropped out of the study after having smoked two joints (once his own weed and once Bediol®). His refusal to further participate was not linked to the Bediol® offered to him or to the aim of the study.

### Data collection

Baseline characteristics included demographics, medication use, use of (illicit) substances (including frequency of cannabis use), motivation to smoke cannabis (specifically as a way to suppress positive and negative symptoms), cannabis craving (Questionnaire on Smoking Urges; QSU-Brief), psychotic-like experiences (CAPE-42), anxiety and depressive symptoms (HADS), and quality of life. It should be noted here that the QSU was developed and tested for tobacco smoking in adolescents and that no reliability and validity data are present for cannabis use and for the current population of patients with a psychotic disorder.

At 15, 60, and 120 min post-smoking, patients completed a questionnaire about their current mood (Profile of Mood States: POMS) [16], and their appreciation of the joint just smoked. For the assessment of the appreciation of the joint, we specifically developed a self-report questionnaire with 16 items scored on a 5-point Likert-type scale ranging from “not at all” to “very much.” Examples of these 16 items are “I like this weed,” “Strong effect,” “Strong kick,” “Feeling stoned,” “Nice taste,” and “I want this weed again.” At 120 min post-smoking, the patient was also asked to express (free-text) his opinion about the cannabis just smoked and whether he would be prepared to change in the future from smoking his own cannabis to this cannabis type.

## Results

The patient’s own cannabis contained 14.0 ± 4.6% *w*/*w* THC and 0.2 ± 0.1% *w*/*w* CBD, which compares well with the strength of the most popular cannabis variants sold in Dutch coffee shops [17]. Patients smoked on average 5.5 ± 5.0 joints per day, and the mean daily THC dose (mean 170.6 ± 115.8 mg) was linearly related to the cannabis craving score (*R*^2^ = 0.42). CAPE-42 total score was 131 ± 36 and HADS score was 12.6 ± 8.0. All patients (also) smoked cannabis for pleasure.

Side effects after smoking each joint were rarely noticed. Psychosis-like signs were not observed, and some transitory nausea and dizziness were only occasionally reported.

The appreciation scores of the three joints (the patient’s own cannabis and the two medicinal cannabis variants) are shown in Table 1 and indicate that the patients appreciated and accepted the two medicinal cannabis variants, though less than their own cannabis. The CBD-enriched variant which contained only 3.6% *w*/*w* THC (Bediol®) and the almost threefold stronger Bedrobinol® (9.1% *w*/*w* THC) was appreciated to the same extent, though Bedrobinol was experienced as “stronger” with a better “kick.” Similar trends were seen for the other ten items of the questionnaire (data not shown). The consistency of repeated questions like “I like this weed,” and “I want this weed again” was high (*r* = 0.96) indicating good reliability. Based on the scores given for “I like this weed” and “Want this weed again,” only limited differences were reported across the three cannabis types tested, whereas the strength and taste of both medicinal cannabis variants were less appreciated than the patient’s own cannabis.

**Table 1:** Sensory effects reported at 15, 60, and 120 post-smoking for “Own cannabis,” Bediol, and Bedrobinol. Scores presented are the mean values obtained in two separate sessions. In parenthesis: percentage of score with own weed as reference

	I like this weed	Strong effect	Strong kick	Stoned feeling	Nice taste	Want this weed again
15 min						
Own weed	2.32	3.23	1.91	2.95	3.18	2.32
Bediol	2.50 (108%)	2.05 (63%)	1.91 (100%)	2.55 (86%)	2.32 (73%)	2.45 (106%)
Bedrobinol	2.55 (110%)	2.32 (72%)	1.95 (102%)	2.41 (82%)	2.09 (66%)	2.27 (98%)
60 min						
Own weed	2.32	2.68	1.82	2.68	2.82	2.27
Bediol	2.00 (86%)	1.64 (61%)	1.50 (82%)	1.91 (71%)	1.64 (58%)	2.41 (106%)
Bedrobinol	2.00 (86%)	1.91 (71%)	1.68 (92%)	1.95 (73%)	1.91 (68%)	1.82 (80%)
120 min						
Own weed	2.27	2.73	1.45	2.36	2.59	2.14
Bediol	2.23 (98%)	1.36 (50%)	1.09 (75%)	1.82 (77%)	1.82 (70%)	1.95 (91%)
Bedrobinol	2.00 (88%)	1.82 (67%)	1.55 (107%)	1.73 (73%)	1.95 (75%)	1.95 (91%)

In the patients’ opinion (given free-text), the two medicinal variants were “Okay,” but again, the major complaint was the low strength and—less so—the less nice taste of the two medicinal cannabis variants. Finally, all patients were asked about their willingness to replace their own cannabis (HPC) by one of the two medicinal cannabis variants, but no clear answers were obtained. However, they expressed that a possible transition would heavily depend on experiences during prolonged use and the options for financial reimbursement.

## Discussion

The main finding of this pilot study among 11 daily cannabis-smoking male patients with schizophrenia was that they did not refuse to smoke the two medicinal cannabis variants as an alternative for their own cannabis, although on average, they preferred the cannabis they usually smoked (own cannabis). As such, a larger and prolonged study aiming to convert patients with a psychotic disorder from smoking HPC to cannabis variants enriched in CBD and/or containing less THC is promising and feasible. Probably, cannabis offered to outpatients as an alternative to street cannabis should contain at least 4% of THC (based on 0.25 g of cannabis per joint). This is supported by a previous study showing that patients with a psychotic disorder did not accept cannabis containing only 0.4% *w*/*w* THC as an alternative [18]. Obviously, quitting from smoking cannabis would be the best option for patients with a psychotic disorder. However, if patients cannot be motivated to quit cannabis use, continued smoking of cannabis containing less THC (and/or more CBD) may result in lower relapse rates, shorter hospitalization, and better psychological functioning [8]. Note that for patients motivated to stop cannabis use, the “milder” cannabis variant might have been a constructive intermediate step in their process to quit smoking cannabis and would express a higher appreciation of Bediol than their non-motivated counterparts.

A clear limitation of the study was that patients did not smoke the two variants over a prolonged time period, but only twice. Another limitation of this pilot study is that patients in the current study were not blinded to their own cannabis. However, blinding of own cannabis would have been redundant because of its typical smell which is easily recognized by the patient. A third limitation is the absence of any psychotic symptoms during the study indicating that the patients included were well treated with antipsychotic medication. For medical ethical reasons, it is, however, impossible to discontinue their antipsychotic medication. A fourth limitation is all patients were male so that it remains to be established whether female patients would show a similar outcome. Finally, just like tobacco smokers, cannabis users may just be accustomed to their own “brand.”

The patients included in this study may be defined as dual diagnosis psychiatric patients with schizophrenia and a substance use disorder. This group of patients usually has a long track of failed treatments, are treatment resistant, have a relatively low quality of life, and are clinically unstable. This group of patients would probably benefit in terms of mental health and well-being if they would use one of the two medicinal cannabis variants tested in the current study. A risk factor for successful cannabis substitution in these patients is that they purchase additional HPC in the coffee shop and smoke it concomitantly with the medicinal cannabis that is prescribed as substitution treatment and thus reimbursed.

In conclusion, our results demonstrate that daily cannabis-smoking patients with a psychotic disorder seem willing to accept smoking cannabis that contains less THC and/or more CBD as an alternative for the HPC they usually smoke. As such, attempts to transfer such patients from smoking HPC to less harmful cannabis variants over a prolonged period of time seems promising and feasible.

## Abbreviations

CAPE: Community Assessment of Psychic Experiences
CBD: Cannabidiol
HADS: *Hospital Anxiety and Depression Scale*
HPC: High potency cannabis (“skunk”)
THC: Δ9-tetrahydrocannabinol
